# Unofficial Media, Government Trust, and System Confidence Evidence From China: An Empirical Exploration of the Attitudes of Netizens Based on the Dual Moderating Effect

**DOI:** 10.3389/fpsyg.2021.763658

**Published:** 2022-01-14

**Authors:** Caijuan Chen, Li Li, Jie Ye

**Affiliations:** ^1^School of Marxism, Hangzhou Normal University, Hangzhou, China; ^2^School of Public Affairs, Zhejiang University, Hangzhou, China; ^3^Department of Sociology, London School of Economics and Political Science, London, United Kingdom; ^4^School of Public Administration, Zhejiang Gongshang University, Hangzhou, China

**Keywords:** political communication effects, unofficial media use, system confidence, official media use, government trust

## Abstract

Mass media has a significant impact on public support for the government. This manuscript constructs a mixed model with official media use as the moderating variable and government trust as the intermediary variable to explore the mechanism of how unofficial media use affects system confidence, using data from a survey of the political and social attitudes of netizens (2015). The study finds that official media use weakens the negative role of unofficial media use in building system confidence, with the intermediary variable of government trust creating the necessary conditions for weakening the effect of unofficial media use. Moreover, the effect of unofficial media use on system confidence is heterogeneous. These findings remind us that it is necessary to deepen research into the micromechanisms that explain how unofficial media use reduces system confidence, a task for which cognitive theory is well suited.

## Introduction

Political and mass media scholars believe that “the public” is in fact a constructed concept (Srinivasan and Diepeveen, 2018). These authors point out that the media not only shapes the public’s perception of political philosophy, such as “what justice is” (Baranauskas and Drakulich, 2018), but also influences the public’s understanding of public policy issues in various ways (Woodruff, 2019); these influences exist in the processes of agenda-setting, policy development, evaluation, and termination (Fawzi, 2018). Due to these influences, the media is seen as playing a central role in democratic politics (Arceneaux et al., 2016). Studies show that media reports have played a promoting and polarizing role (Han and Wackman, 2017; Wojcieszak et al., 2017) on topics including immigration and border control policies (Van Klingeren et al., 2017; Wenzel and Żerkowska-Balas, 2019), ethnic and religious identity (Shaver et al., 2017; Fang et al., 2019), climate politics (Thaker et al., 2017; Rhomberg et al., 2018), the Euro crisis (Rothmund et al., 2017), populism (Muller et al., 2017), bill amendments (Melenhorst, 2015), protest movements (Jost et al., 2018), and electoral politics (Neiheisel, 2016). Even the Roman Catholic Pope has learned to use social media to address political problems (Genovese, 2019). Moreover, large tech companies such as Google, Facebook, and Amazon, which have a vast consumer base, are believed to be capable of manipulating the public’s emotions, promoting certain ideological positions, and influencing people’s behavior (Langlois and Elmer, 2019). All in all, political discourse has become increasingly “mediumized” (Kissas, 2018).

Since the 1970s, three key theories regarding the effect of mass media on political communication have developed: media depression theory (Robinson, 1976; Patterson, 2011), benign-cycle theory (O’Keefe, 1980; Wolfsfeld et al., 2016; Bucy and Groshek, 2018; Dvir-Gvirsman et al., 2018), and media neutralism (Hu and Zhuang, 2017). Interestingly, it is that media neutralism holds that when thinking about the media’s effect on political communication, we should pay attention to the different attributes of the media and the differentiated effects of these attributes on political communication rather than focusing on generalized concepts and symbols (Kaid and Johnston, 1991; Reichert and Print, 2017). Based on this, western scholars have gradually reached the conclusion that different media outlets, content, usage frequency, and levels of trust in different sources of media all have different effects on political communication, including differences in effect size and even effect directionality (Feldman, 1995; Wilkins and Bates, 1995; Miller and Krosnick, 2000; Banducci et al., 2017; Savoy, 2018).

China’s media system has its own unique characteristics (Lee et al., 2018) that differ from those of western media. Against the background of CPC’s propaganda system, the relationship between the news media and the public’s political trust is likely to differ from that in western countries. Accordingly, this manuscript introduces political trust and unofficial media use as intermediary and moderating variables, respectively, to deepen the study of political media communication and the effect of unofficial media use. Specifically, this manuscript makes the following contributions to existing research: (1) we focus on the indirect effects of the independent variable and the interaction effect with other variables by concentrating on the direct and net effects of the independent variables affecting political trust; (2) by taking official and unofficial media use into the analytical model “simultaneously” but not “side by side,” and by analyzing the mediated moderator, we expand the research work in the field of political communication; (3) based on exciting extant research (Easton, 1965; Muller et al., 1982) and actual research data, we divide the political trust into system confidence and government trust, with the help of moderating effect analysis, and further comprehensively examine the contingency mechanism and preconditions to changes in system confidence. In this study, we try to answer the following questions in the Chinese context: Can a higher frequency of unofficial media use bring about lower levels of system confidence? If so, can the use of official media “catalyze” the negative impact on system confidence caused by unofficial media use? If the answer is still affirmative, then the next question will be the following: in the context of the negative influence of unofficial media use on system confidence, is the intermediary role of government trust a necessary condition for the catalytic role of official media use?

### Theoretical Assumptions

#### Impact of Unofficial Media Use on System Confidence

In essence, “unofficial media” refers to a medium of information dissemination that does not need to deliberately spread an official message actively realize the will of the government, or take on the role of propagating the party and the state’s principles and policies. In reality, unofficial media covers information transmission channels ranging from “We-media” (sometimes referred to as “citizen media,” “personal media,” or “private media”), overseas media, and interpersonal verbal communication (Neiheisel and Niebler, 2015). Lu (2008) notes that the formation, development, and influencing of public opinion are processes in which the psychology and behavior of multiple participants are constantly changing, characterized by interdependence, interplay, and mutual constraint; these are also the processes in which multiple participants constantly compete for power and influence over public opinion.

The “system” is a multidimensional concept. This article focuses on the political system; that is, the term herein refers to the socialist political system with Chinese characteristics. Self-confidence refers to positive self-affirmation and self-confirmation among individuals (Che, 2002). Introducing this concept to system analysis, system confidence can be used to describe the extent to which the subject recognizes, affirms, and confirms the system in which they live (Zhang and Yi, 2013). The subject of system confidence can be either the constitutor or the constraint objects; this manuscript will focus on the latter. Therefore, system confidence can be defined as people’s recognition, affirmation, and confirmation of the socialist political system with Chinese characteristics. System confidence depends on two aspects: objective system performance and subjective self-evaluation (Gao, 2015). Evidently, subjective self-evaluation has a more direct impact on system confidence. The variability and uncertainty of subjective evaluation, along with its inconsistency and nonequivalence with objective performance, determine the important impact of external conditions and external factors (such as news media) on system confidence.

Existing studies show that obtaining political news from online communities, a typical form of unofficial media, increases critical attitudes toward those in power, causing a negative impact on satisfaction with and trust in the current political system, and even raising the level of political opposition (Lee, 2015; Lee et al., 2015; Bucy and Groshek, 2018). In fact, the negative political significance of unofficial media is global (Ma and Sun, 2014). The intrinsic mechanism of this negative impact is that Weibo and other online communities, as public forums, actually provide a platform for the public to discuss political information, express political anger, and thus lose political confidence (Russmann, 2018). Such platforms bypass the “prism” of traditional media (Fisher et al., 2018; Garland et al., 2018), thereby distracting from and threatening traditional media’s agenda-setting ability (Feezell, 2018). Simultaneously, the characteristic human pursuit of content pertaining to political scandals and social injustices, in conjunction with the highly entertaining and even commercialized media development models in play (Xia, 2000), provides avenues for unofficial media to disseminate negative social and political news, thereby undermining political trust. Furthermore, the government supervision model cannot cope with the massive and growing amount of social and political news information obtained by the public from unofficial media, which provides the institutional possibilities for the negative impact of unofficial media on system confidence (Ariely, 2015; Zhang and Lin, 2018). More critically, access conditions (minimal barriers to entry, zero associated costs), rapid replication, information distortion and fabrication, users’ inherent ability to comment on provided content, and lack of oversight overreporting methods (Cushion, 2018), along with the long-term repetitive and cumulative effects thereof (Gvirsman et al., 2016), provide an expanded mechanism for the negative influence of unofficial media on system confidence (Hu, 2002). Accordingly, we propose the following hypothesis:

***Hypothesis 1:*** *Unofficial media use has a negative effect on system confidence; the higher the frequency of unofficial media use, the lower level of system confidence.*

#### Official Media Use as a Moderator

Will the same level of unofficial media use occurring in individuals with different levels of official media use produce different system confidence effects? If the answer is yes, this indicates that official media use affects the role of unofficial media use in system confidence. So, what are official media? We contend that official media are those avenues of information dissemination that are required to deliberately spread official narratives, actively realize the will of the government, or take on the role of propagating the party and the state’s principles and policies. Therefore, considered realistically, the media that are directly organized by the government, or mainly express the will of the government and transmit its voice, all belong to the category of official media. Accordingly, the People’s Daily, CCTV, and other government media outlets, along with Netease, Phoenix, Sina, Sohu, and similar large portals^1^, can be classified as official media. According to Bourdieu (1971)’s field theory, official media has the power attribute; specifically, this theory contends that as society develops, fierce violence and material oppression are increasingly deployed in service of the successful implementation of power, meaning that the “symbolic power” of official media becomes increasingly important (Swartz, 2012). Therefore, in terms of its deep meaning, official media and the narratives it shapes is a structural space based on political factors and an important tool used by the political system to realize its will (Rui and Chen, 2013).

As mentioned above, the official media’s essence and mission are to support the existing regime and the achievement of its goals. Any news reports or public opinion that impedes the official will, damages official benefits, or weakens the legitimacy of the regime is the “struggle object” of official media. Consequently, the knowledge and information disseminated *via* unofficial media that is not supportive of the existing political system also represents such a struggle object for official media. To carry out an effective struggle, official media must seize and firmly grasp the ideological leadership; that is, exercise influence over the political discourse and information control power, including the rights of asking, judgment, interpretation, and criticism (Gainous et al., 2018). Therefore, when unofficial media disseminates information that is not conducive to the existing regime and reports news detrimental to the existing system, official media will stand up for the first time, take the form of institutionalized and organized propaganda machine, rely on authoritative and professional media mechanisms, and utilize “taming” mechanisms both open and concealed (Lams, 2018), such as analyzing critical television news programs (Pasitselska, 2017), expressions of political opinion and online criticism (Hyun and Kim, 2015), and also high-intensity propaganda deployed over a short period of time (Huang, 2018), to clarify, explain, criticize, and crack down on these adverse news reports, thereby weakening the negative impact of unofficial media use on system confidence. Accordingly, we propose the following hypothesis:

***Hypothesis 2:*** *Official media use has a positive effect of moderating the relationship between unofficial media use and system confidence.*

#### Official Media Use as a Mediated Moderator

Based on Hypothesis 2, we further examine whether, in the process of unofficial media use impacting system confidence, the positive moderating effect of *official media use* is based on government trust.

Trust refers to the recognition of and dependence on the character and ability of a person, institution, etc (Zhang et al., 2016). When considering the government as the object of trust, it can be readily concluded that government trust^2^ can be understood as the citizens’ belief or confidence that the outcomes produced by the government or the political system are consistent with their expectations (Easton, 1965); that is, citizens believe that the political subject can act for their benefit, or the political subject has the ability to provide appropriate political products to citizens and political communities (Wang, 2016) that actually reflect the citizens’ assessment of the government authority and the political institutions’ performance (Corrigall-Brown and Wilkes, 2014). Norris (1999) also separates the “government” element of “government trust” into the political community, the political system implemented by the political community, and political actors. In this study, government trust is defined with reference to the third level; that is, it refers to the citizens’ trust in the behavior of political actors (the government institutions and their staff) and is a result of specific satisfaction with outcomes based on one’s own interests that are proposed, will be proposed, or have been proposed by the citizens and provided by political actors (Easton, 1965).

This manuscript proposes that government trust plays an intermediary role in the moderating effect of official media use. The inherent logic is as follows: in the face of the antigovernment, antiinstitutional tendency of information on unofficial media and its destructive effect on system confidence, any direct, boring, and empty preaching by official media (along with “prohibition of publication,” “refutation of rumor,” and other crude administrative orders targeted at unofficial media) will be useless or invalid in the process of competing for, maintaining, and improving the ideological leadership and discourse power. The effective approach would be for official media to supply specific and emotional information: for example, disseminating positive descriptions and interpretations of the actual government institutions and their staff, making open and sincere apologies for the objective existence of chaos and inaction among government institutions and their staff, taking information management and control measures and responding in a timely fashion, effectively refuting and resolutely cracking down on exaggerated, inaccurate or even false reports pertaining to government institutions and their staff, taking advantage of professional teams, professional organizations, professional theories and authorities, authoritative voices, and authoritative channels (Wang and Shen, 2017), setting out facts and reasoning things out, and thereby gaining the understanding and support of the citizens and demonstrating the government’s strength in its capacity to maintain social control and political order (Huang, 2018; Bulovsky, 2019). Only in this way, we can truly achieve the goal of weakening the negative impact of unofficial media use on government trust and thus exert a positive influence on system confidence. Accordingly, we propose the following hypothesis:

***Hypothesis 3:*** *The moderating role of official media use in the relationship between unofficial media use and system confidence is based on the intermediary role of government.*

**FIGURE 1:**
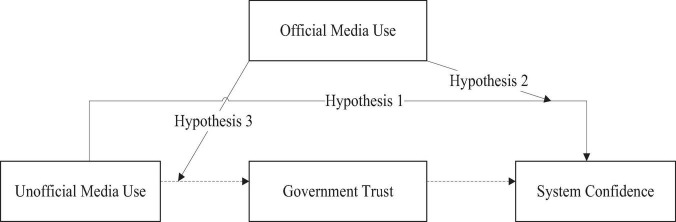
Theoretical model.

The theoretical model used in this study is illustrated in Figure 1.

### Methodology

In this section, we provide an overview of the sample, measurement, and analytical techniques employed in this research.

### Sample

The data of this study are derived from the ‘‘Survey on the Political and Social Attitudes of Netizens ((SPSAN),’’ which can be downloaded at the bottom of the second page^3^. To understand the specific situation as regards the social consciousness of the general public in China, Professor Ma D.Y. of China’s Renmin University has carried out online surveys of Chinese netizens by publishing online questionnaires on the Internet since 2012. The questionnaire includes the respondents’ demographic variables, political psychology, political cognition, and political attitudes. Five such large-scale online surveys were conducted in 2012, 2013, 2014, 2015, and 2017, respectively. The data used in this manuscript are drawn from the 2015 edition. Due to research and technical limitations, only netizens on the Sina-microblogs, WeChat, and Wenjuan (questionnaire site^4^) platforms were surveyed. Given the coverage and influence of Sina-microblogs, however, the data we obtained are highly representative and typical. Samples with only senior middle school education and below are defined as the basic education group, those 30 years of age and below are defined as the junior group, and those with family income of less than 60,000 Yuan are defined as the low-income group; other samples are sequentially defined as the high-education group, senior group, and high-income group, respectively. The relevant descriptive statistics of the variables are shown in Table 1.

**TABLE 1:** Sample distribution.

	Gender (avg/sd) 0.710/0.454		Income (avg/sd) 6.946/2.173		Party member (avg/sd) 0.256/0.438		Education (avg/sd) 3.054/0.633		Age (avg/sd) 2.926/1/226	
Group	Male	Female	High	Low	Yes	No	Basic	High	senior	junior
Variable	2554	1041	2470	1125	932	2663	424	3171	2169	1426
Official media use (avg/sd)	2.633/0.986	2.798/0.964	2.736/1.022	2.561/0.881	2.874/1.024	2.613/0.960	2.444/0.876	2.713/0.993	2.627/1.020	2.763/0.920
Unofficial media use (avg/sd)	3.715/0.965	3.665/0.980	3.751/0.972	3.589/0.955	3.782/1.043	3.672/0.941	3.557/0.941	3.719/0.972	3.742/0.972	3.636/0.963
System confidence (avg/sd)	2.578/1.277	3.329/1.088	2.784/1.268	2.820/1.278	3.029/1.261	2.713/1.265	2.602/1.293	2.821/1.266	2.550/1.301	3.168/1.126
Government trust (avg/sd)	2.489/0.982	3.007/0.897	2.628/0.969	2.662/1.023	2.805/0.955	2.581/0.991	2.463/0.992	2.662/0.983	2.464/1.001	2.904/0.889

### Measurement

In this study, system confidence points to citizens’ recognition and trust of the current major political system: for example, the existence of one party in power with a long-term political strategy^5^ and a noncompetitive electoral system. Government trust refers to the citizens’ level of trust in the government institutions and their staff. Adopting the method of categorization used by Ma and Wang (2016), official media use refers to the consumption of information published in media with an official background, that is officially controlled and supported, or that has taken on the role of reflecting the official will. Therefore, official information use mainly refers to the frequency with which respondents receive political news, current affairs comments, and other news from outlets such as CCTV, Xinhua News Agency, People’s Daily, and Sina Portal. Accordingly, unofficial information use mainly refers to the frequency with which respondents receive political news, current affairs comments, and other news from sources such as We-media, overseas media, and interpersonal verbal communication. Measurements are shown in Table 2.

**TABLE 2:** Measurements.

**Latent variables**
*System confidence (Cronbach’s α* = 0.911)
Electoral democracy is a sham, so China should not engage in it (factor loading: 0.821)
Although the political system of our country has some problems currently, it is still the most suitable system for China’s national conditions (factor loading: 0.902)
Separation of power is not suitable for China’s national conditions. China should never copy the western political system (factor loading: 0.916)
In a democracy, arguments occur every day that will ruin the development opportunities, which make the democracy inferior to the one-party state that has been working effectively for a long time (factor loading: 0.912)
*Government trust (Cronbach’s α* = 0.867)
To what extent do you trust the central government? (factor loading: 0.816)
To what extent do you trust the town government? (factor loading: 0.832)
To what extent do you trust the court? (factor loading: 0.884)
To what extent do you trust the police? (factor loading: 0.857)
*Unofficial media use (Cronbach’s α* = 0.535)
Frequency of obtaining political news, current affairs comments from Weibo or online communities (factor loading: 0.483)
Frequency of obtaining political news, current affairs comments from WeChat communities (factor loading: 0.721)
Frequency of obtaining political news, current affairs comments from unofficial news, or chatting with friends (factor loading: 0.735)
Frequency of obtaining political news, current affairs comments from overseas media (factor loading: 0.645)
*Official information use (Cronbach’s α* = 0.713)
Frequency of obtaining political news, current affairs comments from CCTV news or commentary programs (factor loading: 0.872)
Frequency of obtaining political news, current affairs comments from the political news published by Xinhua News Agency and People’s Daily (factor loading: 0.863)
Frequency of obtaining political news, current affairs comments from the political news published by Sina and other such websites (factor loading: 0.655)

To obtain the specific value of each latent variable, we also factorize and polymerize the items involved in the latent variables. The results show that the KMO values for system confidence, government trust, official media use, and unofficial media use items are 0.840, 0.819, 0.610, and 0.602, respectively. Bartlett’s spherical test shows that the correlation probability between variables is 0.000, which satisfies the factor analysis needs. We completed the factor analysis by means of the principal component method (PCM), using one common factor extracted from the variable items.

Data on the latent variables were determined while we obtained the factor scores. The factor load values of all items exceed the commonly cited value of 0.4, which indicates that items under the same factor can effectively reflect the same construct. The KMO value and the factor load value support the validity of the latent variables. In addition, we treat the demographic variables of gender, age, education level, CPC membership, and household income as control variables in the regression models.

### Models and Analytical Techniques

In this study, we use the hierarchical linear model (HLM) to verify the hypotheses. The regression models comprise control variables, an independent variable, a moderating variable, an intermediary variable, and a dependent variable. All statistical work was performed using SPSS19.0, AMOS17.0, and Stata12.0 when required.

### Empirical Results

The means, standard deviations, and correlations of the latent variables are shown in Table 3. The results reveal that a significant correlation exists between the variables; accordingly, Hypothesis 1 receives initial support.

**TABLE 3:** Descriptive statistics and correlation matrix of latent variables.

Variables	Average	Standard deviation	Official media use	Unofficial media use	System confidence	Government trust
Official media use	2.68	0.984	1.000			
Unofficial media use	3.70	0.970	0.278****	1.000		
System confidence	2.64	0.986	0.546****	−0.052***	1.000	
Government trust	2.80	1.271	0.430****	−0.057***	0.671****	1.000

Two-sided t-tests. ****p < 0.001, ***p <0.01.

### Single-Source Bias Test

In this manuscript, we test the single-source bias of each item by means of Harman’s single-factor detection method. Specifically, we use a single factor to analyze the items on the questionnaires. The results show that, in the factor analysis without rotation, the first principal component can explain the variation in 30.6% of all items, indicating that single-source bias does not affect the reliability of the conclusions.

### Reliability and Validity

In terms of reliability, the results show that the internal consistency coefficients of the four main variables are at a high level (see Table 2). In addition to calculating the KMO value and performing the factor load test, this manuscript also tests the distinction of validity between variables. Four-factor model confirmatory factor analysis reveals that, except for the chi-square value and its significance for absolute fit, all other fitness index indicators are at a high level (see Table 4). The chi-square value is subjected to fluctuations depending on the number of samples. Wu (2013) has noted that, in the case of large samples, the chi-square value will always satisfy the statistical demands; accordingly, the data in our study, coming as it does from a large sample, are valid for statistical analysis.

**TABLE 4:** Model fit calculation results.

Absolute fit			Incremental fit				Parsimonious fit			
χ^2^/df	*P*	RMSEA	NFI	RFI	IFI	TLI	CFI	PGFI	PNFI	PCFI
21.49	0.000	0.076	0.934	0.918	0.937	0.921	0.931	0.654	0.747	0.750

### Heteroscedasticity, Sequence Correlation, and Collinearity Test

Before performing regression analysis, to prevent heteroscedasticity, we chose to run a robust regression in Stata 12.0. At the same time, we performed a Durbin–Watson (DW) test to test the sequence correlation between variables. The results show that the DW values of all equations are around 2, indicating that no sequence correlations exist between variables. We used the variance inflation factor for all models to test for multicollinearity. For all models, the maximum VIF was less than 2, proving that the data do not exhibit obvious collinearity problems.

### Hypothesis Tests

We obtained confirmatory results on all three of our tests.

#### Main-Effect Test

From Table 5, we can see that when we fix the control variables, regression of unofficial media use (the independent variable) yields a regression coefficient of −0.037 when the significance test is used at the 0.05 level (Model 1). Furthermore, the model shows that in addition to household income, control variables such as education, gender, party membership, and age all have significant impacts on system confidence. This demonstrates that unofficial media use can significantly reduce the level of system confidence; thus, Hypothesis 1 is confirmed.

**TABLE 5:** Main effect, moderating effect, and mediated moderating effect.

	System confidence			Government trust	System confidence
	*Model 1*	*Model 2*	*Model 3*	*Model 5*	*Model 7*
Age	−0.279****	−0.203****	−0.201****	−0.159****	−0.131****
Education	−0.028*	–0.020	–0.018	0.005	−0.020*
Gender	−0.214****	−0.180****	−0.179****	−0.166****	−0.105****
CCP membership	0.144****	0.080****	0.080****	0.073****	0.047****
Household income	–0.013	−0.045***	−0.046****	−0.026*	−0.034***
Unofficial media use	−0.037**	−0.191****	−0.197****	−0.178****	−0.116****
Government trust					0.361****
Official media use		0.556****	0.547****	0.418****	0.447****
Official media use *unofficial media use			0.029**	0.071****	–0.003
*F*	156.35	535.79	470.36	227.86	722.07
*R* ^2^	0.1642****	0.4377****	0.4384****	0.2930	0.5794****
△R^2^		0.2753****	0.0007**	0.0043****	0.0000

Two-sided t-tests. ****p < 0.001, ***p < 0.01, **p < 0.05, and *p< 0.1.

#### Test of the Effect of the Moderating Variable (Official Media Use) on the Main Effect

**FIGURE 2:**
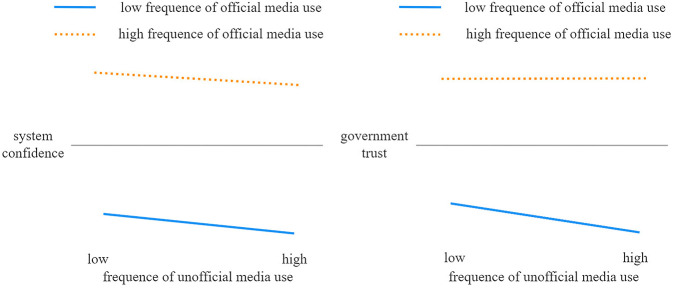
Sketches of the dual moderating effects of official media use.

We adopt the classic three-step test to verify the moderating effect of official media use, employing the interaction between the moderating variable (official media use) and other related variables to test the magnitude and significance of the moderating effect. Specifically, the first step is to verify the influence of the independent variable (unofficial media use) on the dependent variable (system confidence) and its significance. The second step is to examine the influence of the independent variable (unofficial media use) and the moderating variable on the dependent variable (system confidence) and its significance. The third step is to put the interaction term of the independent variable (unofficial media use) and the moderating variable (official media use) into the regression equation. If the regression coefficient of the interaction term is significant and △R^2^ is also significant, this indicates that a moderating effect exists between system confidence and unofficial media use. All these three models include control variables such as education. The empirical results that are shown in Table 5 and Figure 2 confirm the moderating effect.

The requirements of the first three steps have been met. Furthermore, comparing the R^2^ of Model 3 with that of Model 2 reveals that the coefficient of △R^2^ is significant (0.0007), revealing that official media use has a positive moderating effect on the relationship between unofficial media use and system confidence. Hypothesis 2 is therefore confirmed.

#### Test of the Effect of the Intermediary Variable (Government Trust) on the Moderating Effect

In accordance with the hypothesis on mediated moderating effects, we follow the recommendations and judgment criteria of Muller et al. (2005) and perform the following tests in order. First, we determine the regression equations between the dependent variable (system confidence) and the independent variable (unofficial media use), the moderating variable (official media use), and their interaction terms. The coefficient of the interaction term is required to be statistically significant. Second, to make the interaction coefficient statistically significant, we determine the regression equations between the intermediary variable (government trust) and the independent variable (unofficial media use), the moderating variable (official media use), and their interaction terms. Third, to satisfy the requirement that the coefficient of the intermediary variable (government trust) be statistically significant, we determine the regression equations between the dependent variable (system confidence) and the independent variable (unofficial media use), the moderating variable (official media use), the intermediary variable (government trust), and the interaction term of unofficial media use and official media use. In the third step, if the coefficient of interaction term decreases but remains significant, this indicates that the moderating effect works through the intermediary variable (government trust). If the coefficient of the interaction term becomes no longer significant, it proves that the moderating effect works entirely through the intermediary variable (government trust). All these three models include control variables such as education. The empirical results are presented in Table 5

Obviously, the requirements of the first two steps have been met. In the third step, the coefficient of the intermediary effect of government trust on system confidence is also found to be significant. Moreover, the coefficient of the effect of the interaction between unofficial media use and official media use on system confidence changes from being significant in the first step to not being significant thereafter. This shows that the moderating effect of official media use between unofficial media use and system confidence is entirely based on government trust.

With the aim of further evaluating the moderating effect of official media use, we additionally follow the method developed by Aiken et al. (1991) to graphically represent the relationship between unofficial media use and system confidence, in the sense of the main effect, and the relationship between unofficial media use and government trust, in the sense of a mediated moderating effect (see Figure 2). The left-hand part of Figure 2 shows that unofficial media use has a negative effect on system confidence regardless of whether the degree of official media use is high or low. However, it is also obvious that the slope of the sample with higher official media use is smoother than that of the sample with lower official media use; that is, unofficial media use has a more negative influence on system confidence when people have lower levels of official media use. Moreover, the right-hand part of Figure 2 shows that whether official media use level is high or not, unofficial media have a negative impact on government trust in the form of a mediated moderating effect, and the slope of the group with lower official media use is steeper. This shows that the negative influence of unofficial media use on government trust among people with higher levels of official media use is relatively weak.

### Heterogeneity Analysis

To further analyze the influence of unofficial media use on system confidence, and also the mechanism of that influence, we conducted a heterogeneity analysis. The results are reported in Table 6.

**TABLE 6:** Heterogeneity analysis estimates.

		High income	Low income	Female	Male	Senior	Junior	Basic education	Higher education	Party-member	Non-party
	Group	(1)	(2)	(3)	(4)	(5)	(6)	(7)	(8)	(9)	(10)
Main effect	Effect	Not exist	Exist	Not exist	Exist	Exist	Not exist	Exist	Not exist	Not exist	Exist
	Control variable	Yes	Yes	Yes	Yes	Yes	Yes	Yes	Yes	Yes	Yes
	Regression coefficients	0.015	−0.095****	−0.004	−0.054***	−0.074****	0.013	−0.205****	−0.024	−0.043	−0.040**
Moderating effect	Effect	/	Exist	/	Not exist	Exist	/	/	/	/	Exist
	Control variable	/	Yes	/	Yes	Yes	/	/	/	/	Yes
	Interactive variable significance	/	0.037**	/	0.013	0.032*	/	0.071*	/	/	0.026*
	△R2 significance	/	0.001**	/	0.000	0.001*	/	0.005*	/	/	0.001*
Mediated moderating effect	Effect	/	Not exist	/	/	Exist	/	Exist	/	/	Exist
	Control variable	/	Yes	/	/	Yes	/	/	/	/	Yes
	Intermediary variable significance	/	0.450	/	/	0.437****	/	0.480****	/	/	0.455****
	Interactive variable significance	/	−0.007	/	/	−0.002	/	0.017	/	/	−0.005

Two-sided t-tests. *p < 0.1; **p < 0.05; ***p <0 .01; ****p < 0.001.

### Income Heterogeneity

As Table 6 shows, unofficial media use significantly reduces the system confidence of the low-income group. This is in line with findings from other researches. As one study using four provinces in China as samples shows, people with lower monthly household incomes have poorer overall cognitive abilities (Gao et al., 2021) and very readily believe information provided in unofficial media. High-income groups have stronger cognitive abilities, meaning that their results are different. Their use of nonofficial media has no substantial impact on their system confidence.

### Gender Heterogeneity

The greater the audience’s exposure to social media, the easier it is for them to perceive the many advantages of social media and increase their trust in its content (Jackob, 2010; Xue et al., 2014). This article draws the same conclusion. Moreover, different from the male group, as Table 6 shows, unofficial media use does not substantially change the level of system confidence for the female group. This is also in line with the findings of existing research: Chinese women’s points of attention are mainly distributed across daily life, family, and work (Zhou et al., 2018). For them, the negative social news and political reports they encounter on unofficial media may simply be passively observed and then dismissed in an instant; thus, their unofficial media use does not meaningfully reduce their trust in the political system.

### Age Heterogeneity

Empirical research shows that older people have higher media literacy and comprehension ability (Jones-Jang et al., 2021). The results of this manuscript support this view. As can be seen from Table 6, unlike the senior group, unofficial media use does not substantially affect the level of system confidence among the junior group. One possible explanation for this phenomenon is that due to their young age and limited experience, this group is easily affected by the media, but their perception of social affairs is unstable.

### Educational Heterogeneity

People with higher levels of political knowledge are more convinced of their opinions without being affected by other media frameworks (Kinder and Sanders, 1990). People with higher levels of education also have a higher level of political knowledge. It can therefore be inferred that groups with higher education are not as easily influenced by nonofficial media. As Table 6 shows, for the higher education group, unofficial media does not meaningfully reduce their recognition and trust in the political system. The basic education group is different. For this group, the higher their frequency of unofficial media use, the more negative social news they receive, and the more easily they lose their recognition of and trust in the political system.

### Political Appearance Heterogeneity

It can be seen from Table 6 that unofficial media use does not substantially reduce system confidence for the party-member group. One possible explanation is that the party-member group has more political trust (Chen and You, 2021), which prompts them to dismiss unofficial media and the negative social and political news it presents. However, the nonparty group has a different outcome. Because of the “nonmainstream” nature of their political beliefs and political identity, this group is more likely to believe in unofficial media and the negative social news it provides.

## Conclusion

In recent research, scholars have found that mass media influences and shapes political attitudes, judgments, interests, emotions, and culture, and also political knowledge (Adena et al., 2015; Kim, 2017; Lecheler and de Vreese, 2017; Chang, 2018; Kwak et al., 2018; Lee, 2018; Lee et al., 2018; Titifanue et al., 2018; Xia and Shen, 2018). The characteristics of China’s media system differ from those in western countries (Lee et al., 2018). Against the background of the party’s propaganda system, the relationship between the news media and the public’s political trust in the Chinese context is complex, specific, and characterized by a certain degree of uncertainty, meaning that further exploration is required. Accordingly, in the context of today’s increasingly complex media environment (particularly given the current pandemic situation), exploring the impact of official and unofficial media on government trust and system confidence through a variety of research paths is of great value for enriching and expanding the field of political psychology. The conclusions of this manuscript are summarized below.

First, official media use has a reverse catalytic effect on the political deconstruction function of unofficial media use. This is in line with the media depression theory (Robinson, 1976) stating that news media not subjected to government control has its own antigovernment and antisystem attributes. However, this does not mean that official media can do nothing or must necessarily “surrender.” In many cases, unofficial media has the advantage of being able to publish quickly. However, if official media is able to subsequently report, explain, and clarify in a timely, truthful, and candid fashion (especially in emergencies), do a good job of dynamically tracking reports and inform the public as to the progress of the incident and the specific measures being taken by the government, it can play an effective role in weakening and suppressing the antisystem and antigovernment attributes and capabilities of unofficial media.

Second, the political construction function of official media has a precondition: the political identity logic of the public has a significant pragmatic tendency. From the cognitive perspective of primacy effects (Luchins, 1957), the information one encounters first has a stronger effect on one’s subsequent interpretation of events. Unofficial media have a temporal advantage in reporting on news events and take priority based on this; thus, negative reports and negative interpretations, especially those that operate by directly or metaphorically attributing these negative events to the functioning of the political system, will effectively reduce the public’s trust in and recognition of political systems. However, as discussed above, official media can still make a difference in these cases, although it should be noted here that the “antiattack” and “enhancement” roles of official media must be based on the intermediary mechanism of government trust.

Third, the heterogeneous unofficial media’s political communication mechanism only beginning to be understood. From the subsample data, the effects of the official media’s political communication on different groups are varied. Therefore, it is very complicated and difficult to achieve the purpose of restraining and weakening the negative impact of unofficial media on system confidence through official media channels.

This article remains subject to certain limitations and can be further improved. First, due to the impact of objective conditions, this study relies on surveys of netizens, which limits the representativeness of the sample and therefore its broader external validity. In the future, we should add political communication items to the national microsurvey to improve both the representativeness of the sample and the accuracy of our extrapolated conclusions. Second, the Cronbach’s α score of the four items related to unofficial media is only 0.535, which represents a key limitation of the study. Since these four media platforms are typical examples of unofficial media in the Chinese context, both in theory and in practice, this article has opted to include all of them. However, the factor analysis of these four items produces only one common factor, and the factor loads of the four items all exceeded 0.4; this indicates that they all point to only a single factor, which supports the decision made in this article to maintain these items to a certain extent. This issue merits further study in the future. Third, since this is a study in the context of China, the items that constitute system confidence are primarily oriented to China’s political reality. Therefore, the generalizability of these results to other countries represents an issue. In the future, the items related to system confidence should be made more universal to improve the generalizability of the research. Fourth, because we were unable to conduct multistage and multivariable tracking investigations on our sample, the data analysis in this manuscript is necessarily based on the correlations between variables alone; thus, it cannot rely entirely on statistical results to judge the causal relationship, and there may be endogeneity problems. Although, in the process of hypothesis construction, this study has theoretically demonstrated the specific one-way relationship among the independent variable, intermediary variable, and dependent variable, a suitable instrumental variable should also be identified, cognitive-theory tools employed, and qualitative research undertaken to back up our findings regarding the role played by unofficial media use in reducing system confidence.

## Data Availability Statement

The original contributions presented in the study are included in the article/supplementary material, further inquiries can be directed to the corresponding author.

## Ethics Statement

Ethical review and approval was not required for the study on human participants in accordance with the local legislation and institutional requirements. Written informed consent for participation was not required for this study in accordance with the national legislation and the institutional requirements.

## Author Contributions

CC contributed to the experimental design, execution of the experimental research, completed the data analysis, and wrote the first draft of the manuscript. LL participated in the experimental design and analysis of the experimental results. JY contributed to the project design, was the person in charge, and guided experimental design, data analysis, thesis writing, and revision. All authors have read and agreed to the final text.

## Conflict of Interest

The authors declare that the research was conducted in the absence of any commercial or financial relationships that could be construed as a potential conflict of interest.

## Publisher’s Note

All claims expressed in this article are solely those of the authors and do not necessarily represent those of their affiliated organizations, or those of the publisher, the editors and the reviewers. Any product that may be evaluated in this article, or claim that may be made by its manufacturer, is not guaranteed or endorsed by the publisher.

## Footnotes

1 Because Sina, NetEase, Phoenix, and other such large portals have a huge audience and a strong public opinion guidance function, they are highly valued and controlled by the party’s propaganda department. Judging from the actual situation in China, the political news reports published by these portals must meet the requirements of the party and the government. When it comes to political news specifically, these portals must reflect the will of the party and government and guarantee support for their interests. Accordingly, in this article, we regard these large portals’ political news as “official media.”

2 Some domestic scholars equate government trust with political trust; see for example, Hu and Zhuang (2017). We define government trust from the perspective of the political system; that is, herein, government trust is equivalent to political trust.

3
http://cnsda.ruc.edu.cn/index.php?r=projects/index&Projects_page=2

4
www.wenjuan.com

5 The report of the 19th CPC National Congress clearly states that the leadership of the CPC is the most essential feature of socialism with Chinese characteristics.
